# Enhancement of cannabidiol oral bioavailability through the development of nanostructured lipid carriers: In vitro and in vivo evaluation studies

**DOI:** 10.1007/s13346-024-01766-9

**Published:** 2024-12-30

**Authors:** Iman E. Taha, Mahmoud A. ElSohly, Mohamed M. Radwan, Rasha M. Elkanayati, Amira Wanas, Poorva H. Joshi, Eman A. Ashour

**Affiliations:** 1 Department of Pharmaceutics and Drug Delivery, School of Pharmacy, University of Mississippi, University, MS 38677 USA; 2 National Center for Natural Product Research, University of Mississippi, University, MS 38677 USA

**Keywords:** Cannabidiol (CBD), Nanostructured Lipid Carrier (NLC), Hot Homogenization, Oral Bioavailability, In Vitro and In Vivo Evaluation

## Abstract

Cannabidiol (CBD) is a natural product isolated from the *Cannabis sativa* plant that was approved by the United States Food and Drug Administration (US FDA) for the treatment of resistant epilepsy. Despite its therapeutic potential, CBD’s clinical application is limited by its poor aqueous solubility and low oral bioavailability. The primary aim of this research was to enhance the aqueous solubility and oral bioavailability of CBD by developing nanostructured lipid carriers (NLCs) using conventional hot homogenization method (CHH). In the current study, nine CBD NLC formulations were developed through CHH, of which, NLC5 emerged as the most promising formulation, exhibiting high CBD entrapment efficiency (99.23%), particle size of 207 nm, a polydispersity index of 0.19, and a zeta potential of -26 mV. Additionally, drug release testing for NLC5 showed a high CBD release rate of more than 90% within 15 min, indicating an enhancement of CBD dissolving rate compared to pure CBD. The in vivo pharmacokinetic study of NLC5 formulation showed 27% CBD oral bioavailability. Furthermore, Stability studies conducted at 4 °C and 25 °C on this formulation over three months, revealed consistent parameters, underscoring the robustness of the formulation. In conclusion, the successful formulation of CBD-loaded NLCs resulted in improved CBD release rate, enhanced oral bioavailability of CBD, and maintained stability, making it a promising approach for the effective delivery of CBD.

**Graphical Abstract:**

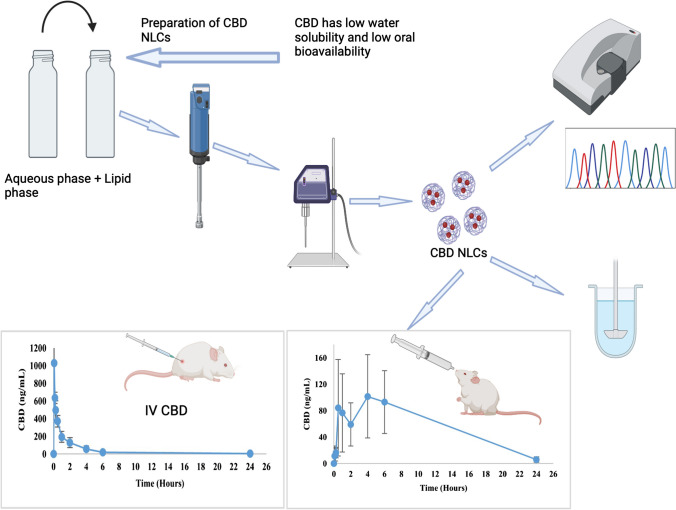

## Introduction

*Cannabis sativa* plant is one of the most ancient crops cultivated for various purposes that was introduced to western medicine during the early 19th century [1]. Among the different active components isolated from *Cannabis*, Δ^9^-tetrahydrocannabinol (Δ^9^-THC) and cannabidiol (CBD) are considered the two major constituents [1]. Because CBD does not have the same psychoactive effects as THC, it is recognized for its safety in the medical field and is known for its diverse pharmacological benefits [2]. These activity included anti-inflammatory, antipsychotic, anti-seizure, and anxiolytic properties [3]. Recently, CBD has shown strong potential in mitigating the side effects associated with cancer therapy [4]. CBD’s immunosuppressive properties have undergone thorough investigation, revealing its ability to inhibit the production of tumor necrosis factor (TNF) and interleukin (IL) cytokines which are involved in inflammation and immune system signaling in both rat and human mononuclear cells [5, 6]. CBD possesses significant lipophilicity (with a log P value of 6.3) and displays poor water solubility (0.1 µg/mL), hence the CBD dissolution is a rate limiting step for its absorption [7, 8]. These characteristics, along with substantial first-pass metabolism, result in low oral bioavailability, estimated at approximately 6% [8]. In addition, research has demonstrated that CBD in liquid formulation is susceptible to degradation triggered by factors such as light, temperature, and air [9]. All these mentioned vulnerabilities present substantial difficulties in the development of CBD formulations. The only available CBD preparation approved by the FDA in 2018 is Epidiolex^®^, which is a 100 mg/mL CBD refined sesame oil-based solution, prescribed for the treatment of seizures caused by Lennox-Gastaut syndrome and Dravet syndrome [10]. Therefore, the rationale behind this study was to develop a formulation for enhancing the release rate and oral bioavailability of CBD.

Improving the solubility and oral bioavailability of poorly water-soluble drugs is a critical challenge in pharmaceutical development. Various physical and chemical methods have been employed to enhance solubility, of which, particle size reduction emerging as a prominent physical technique [11]. Developing nanoparticle formulations, with small particle size, is a key approach adopted to improve drug absorption and oral bioavailability [12, 13]. Among these, lipid-based delivery systems are particularly promising as oral formulations, significantly enhancing the dissolution and oral bioavailability of poorly water-soluble drugs [14]. Upon reaching the small intestine, they undergo in vivo digestion by lipases and co-lipases, transforming into mixed micelles that facilitate drug absorption [15]. The nanosized droplets create a substantial surface area that is stabilized by surfactants, leading to faster dissolution and increased absorption [16]. They also have the potential to enhance oral bioavailability by promoting drug absorption through the lymphatic pathway, and thereby protecting the drug from the initial hepatic metabolism [17].

The nanoparticles formulations can be categorized into two distinct generations, solid lipid nanoparticles (SLN) and nanostructured lipid carriers (NLCs) [12] and they both share similar advantages of being biocompatible, biodegradable and easily scalable for drug delivery applications. However, NLCs offer improved stability and higher drug loading capacity compared to SLNs. This is attributed to the use of both solid and liquid lipids creating more spaces for the drug loading [18, 19]. In the NLCs system, the drug stability is enhanced since it is entrapped inside the liquid lipid and encapsulated by the solid lipid matrix. This structural configuration addresses the issue of drug expulsion during storage commonly associated with SLN formulations [20].

For the development of NLCs, a variety of solid and liquid lipids are used to optimize the formulation, including Dynasan^®^, Precifac, Stearic acid, Compritol^® ^888 ATO, and Precitol^® ^ATO [21, 22]. In conjunction with solid lipids, liquid lipids are used to achieve a partially crystallized system that enhances drug load and stability [23]. Examples of liquid lipids include soya bean oil, oleic acid, medium chain triglycerides (MCT)/caprylic- and capric triglycerides, sesame oil, α-tocopherol/vitamin E, and isopropyl myristate [24]. To ensure the stability of the NLC system, surfactants and co-surfactants are incorporated to prevent aggregation and maintain the homogeneity of the colloidal system [25], which included Tween^® ^20, Tween^® ^80, Lutrol^® ^F68, Lutrol^® ^F127, Solutol^® ^HS 15, Kolliphor^® ^HS 15, and Cremophor^® ^EL [21]. The production of NLCs involves several advanced techniques, each offering unique benefits. High-pressure homogenization is a predominant method and can be conducted through hot or cold processes [26]. Solvent diffusion, ultrasonication, multiple emulsion techniques, and phase inversion are other techniques for preparing NLCs [26].

This study reports on the development of CBD NLCs using CHH to improve the solubility and bioavailability of the drug. Additionally, this study aimed to optimize the formulation of NLCs by selecting ingredients based on their compatibility and suitability for CBD encapsulation. Furthermore, the research focused on characterizing the optimized NLCs formulations, evaluating their physicochemical characteristics, and assessing the bioavailability and the stability of the lead formulation.

## Materials

Tween^®^ 80, sesame oil, and castor oil were purchased from Fisher Scientific (Hanover Park, IL USA). Compritol^® ^888ATO and Dynasan^® ^118 were gifted from Gattefoss´e (Paramus, NJ USA). CBD, with a purity of 99.9% (determined by HPLC), was isolated from a CBD rich cannabis chemovar, cultivated at the University of Mississippi. All solvents used were HPLC grade.

## Methods

### Screening of lipids

The first stage in NLC development involves the careful selection of suitable lipids and surfactants, as they serve as the fundamental building blocks for NLCs formulations [21]. In this study, Compritol^®^ 888 ATO and Dynasan^®^ 118 were selected for evaluation as solid lipids, based on their previously incorporation as solid lipid carriers in NLCs preparation according to relevant literature [27, 28]. The melting temperature of Compritol^®^ 888 ATO and Dynasan^®^ 118 fall within the range of 70 to 75 °C. Consequently, each lipid (200 mg) and an excess amount of CBD were mixed and heated on a hot plate at 80 °C, which is approximately 5 degrees higher than their respective melting points [29]. The mixtures were kept at room temperature for 24 h and visually checked for any precipitation or recrystallization of CBD.

Sesame oil and castor oil were selected as liquid lipids, and their ability to solubilize CBD was carried out visually and analytically [30]. Excess amount of CBD was added to 5 mL of the oil (sesame oil or castor oil) in a glass vial with a cap. The vials were placed on a horizontal shaker (VWR Microplate shaker) and maintained at room temperature for 48 h. The amount of CBD solubilized in the chosen oils were evaluated visually by observing any precipitation of CBD and then the samples were centrifuged at 13,000 rpm for 15 min. The supernatants were analyzed for CBD content using a validated HPLC method [31]. A Luna C18(2) column (150 × 4.60 mm id, 3 μm particle size; Phenomenex, Torrance, CA) was used. The column’s temperature was kept constant at 28 °C. The mobile phase consisted of (A) 0.1% (v/v) formic acid in water and (B) 0.1% (v/v) formic acid in acetonitrile with the following gradient elution program: started and kept at 30% A and 70% B from 0 to 6 min; then to 23% A and 77% B in 6 min; kept at 23% A and 77% B for 10 min; after that, the system was restored to the initial conditions in 0.2 min. The flow rate was maintained at 1.2 mL/min. A five-points calibration curve of CBD was constructed (5, 10, 25, 50, and 100 µg/mL). The regression analysis was performed on the data points, generating the calibration curve of an R2 equal to 0.9999. The Limit of quantitation (LOQ) and Limit of Detection (LOD) of CBD is 4.9 µg/mL and 1.6 µg/mL, respectively.

### Preparation of NLC placeboes, and CBD NLCs

Based on the screening results, Compritol^®^ 888 ATO and Dynasan^®^ 118 were chosen as the solid lipids, sesame oil as the liquid lipid, and Tween^®^ 80 as the surfactant. The NLC placebo was formulated with a ratio of 3:3:4% w/w for solid lipid, oil, and surfactant respectively. Two different placeboes were prepared, one contained Compritol^®^ 888 ATO and the other included Dynasan^®^ 118 as solid lipid. A 10 g batch of each placebo was prepared in a 20 mL scintillation vial. The lipid phase, consisting of the solid and liquid lipids, was melted and mixed at 80 °C in a water bath. Simultaneously, the aqueous phase, consisting of Tween^®^ 80 and distilled water, was mixed and heated to the same temperature in a separate vial. The aqueous phase was added to the lipid phase drop by drop at a stirring rate of 2000 rpm, and the mixture was kept in the water bath for 5 min. Subsequently, the prepared pre-emulsion was homogenized using a T25 digital Ultra-Turrax homogenizer (IKA, Staufen, Germany) for 5 min at 70 °C, cooled to room temperature and then sonicated using a probe sonicator (Vibracell™) for 10 min, with an amplitude of 40%, a pulsing cycle of 10 s on and 10 s off, and a total sonication time of 10 min.

To evaluate the effects of varying the lipids and surfactant concentrations on particle size (PS), polydispersity index (PDI), zeta potential (ZP), and encapsulation efficiency (EE), nine formulations of different lipids/surfactant ratios were prepared with loading of 2% w/w CBD in all NLCs as illustrated in Table 1. CBD was dissolved in the lipid phase before adding the aqueous phase. All the prepared formulations were stored in 20 mL glass vials until further characterization studies.

**Table 1:** Formulations composition of CBD NLCs. Water was added for each composition up to 10 g

Formulation	Sesame oil (%w/w)	Compritol^®^ 888ATO (%w/w)	Tween^®^ 80 (%w/w)	CBD (%w/w)
CBD NLC1	3	3	4	2
CBD NLC2	3	2	4	2
CBD NLC3	2	3	4	2
CBD NLC4	3	3	3	2
CBD NLC5	3	2	3	2
CBD NLC6	2	3	3	2
CBD NLC7	3	3	2	2
CBD NLC 8	3	2	2	2
CBD NLC 9	2	3	2	2

### Evaluation of the developed NLCs

Evaluation and characterization of the developed NLCs involved quantification of important parameters such as PS, PDI, ZP, Drug Content (DC), and Entrapment Efficiency (EE). Dynamic light scattering using a Malvern Zetasizer Nano ZS (Malvern Instruments, UK) was employed to measure PS, PDI, and ZP for the placebo and the nine CBD NLCs. The samples were diluted with distilled water (100x dilution factor) and all measurements were carried out in triplicate at a scattering angle of 90° at 25 °C.

DC and EE of the developed CBD NLCs were evaluated using the previously mentioned HPLC method. Samples (100 µL each) were diluted 400 times with acetonitrile (ACN) and analyzed for CBD content. All experiments were conducted in triplicates. EE quantification involved storing the prepared CBD NLCs overnight, followed by carefully withdrawing a sample from the upper portion avoiding contact with the bottom and walls, to ensure that only the CBD entrapped in the lipid matrix was analyzed. These samples were then diluted with ACN and subjected to HPLC analysis.

### Drug release study

Based on the results from the evaluation studies, the lead formulation with the optimum results (smallest PS, low PDI, and high EE) was selected for further experimentation. The percentage of CBD released from the lead formulation compared to pure CBD was determined using the sample and separate method [32]. This procedure allows the application of samples directly to the dissolution media and then separation of the dispersed nanoparticles from the continuous phase using ultracentrifugation or ultrafiltration technique. The release study was performed using a USP apparatus II under the following conditions: 900 mL of phosphate buffer solution at pH 7.4 + 0.1% Tween^®^ 80, set at a 50-rpm speed, and a temperature of 37 ± 0.5 °C. At each determined time point (15, 30, 45, 60, 90, and 120 min), 1.5 mL sample was withdrawn and centrifuged at 13,000 rpm for a duration of 20 min. HPLC method was used to quantitatively analyze the supernatants for the CBD release.

### In vivo pharmacokinetics studies

Juglar catheterized of the Sprague Dawley rats (200 to 250 g) were used for this study. The rats were divided into two groups, each consisting of four animals, one group received CBD solution intravenous (CBD IV) in a mixture of Cremophor: ethanol: water (1:1:8) and the second group received CBD NLC5 formulation orally. Both groups were housed separately in a controlled laboratory environment. The first group received a dose of 1 mg CBD IV/animal, while the second group received CBD NLC5 (contains 5 mg CBD) /animal. The blood samples (200–300 µL) were withdrawn at 0, 5, 10, 15, and 30 min, and at 1, 2, 4, 6, and 24 h. The plasma samples were collected by centrifuging the blood samples at 3000 rpm for 5 min at 4 °C. The plasma samples were stored at − 80 °C until analysis. All the experimental procedures and protocols have been approved by the Institutional Animal Care and Use Committee (IACUC) of the University of Mississippi under protocol number 22 − 003.

#### Sample preparation

CBD was extracted from the rat’s plasma using a simple protein precipitation method. A 50 µL aliquot of each plasma sample was placed into a 0.5 mL Eppendorf tube. The samples were then quenched with 100 µL of 100% acetonitrile containing an internal standard (20 ng/mL of CBD-d3 or tolbutamide), mixed for 15 s on a cyclomixer (Thermo Scientific, Indianapolis, IN, USA), followed by vortexing for 2 min, and centrifugation for 15 min at 14,000 rpm on an accuSpin Micro 17R (Fisher Scientific, Suwanee, GA, USA) at 4 °C. Approximately 80 µL of the clear supernatant was transferred to LC-MS/MS vials for analysis.

#### Sample analysis

Samples analysis was performed using a reported method [33], a Waters XevoTM TQ-S Acquity UPLCTM system (Waters Corp, Milford, MA, USA) with an autosampler temperature of 10 °C and a column oven set at 40 °C. A Waters Acquity UPLC^®^ BEH Phenyl column (50 mm x 3.0 mm x 1.7 μm) was used for the chromatographic separation, employing a linear gradient elution comprising (A) 90% acetonitrile and (B) 10% 0.2% formic acid in Milli-Q water as mobile phases. The flow rate was maintained at 0.3 mL/min, with an injection volume of 2 µL and a total run time of 5.0 min. For rinsing, a solution mixture of 80% methanol in Milli-Q water was utilized.

An Acquity Tandem Quadrupole Mass Detector (Xevo TQ-S; Waters Corp, Milford, MA, USA) in positive electrospray ionization mode was used for the mass spectrometric detection of CBD while negative electrospray ionization was used for the corresponding metabolites (CBD COOH and OH-CBD. Quantification was performed using multiple reaction monitoring (MRM). The calibration range used for CBD was 5.6–560 ng/mL while the Lowest Limit of Quantification (LLOQ) was 5.6 ng/mL. Similarly, the calibration range used for CBD COOH and OH-CBD was 5.0–500 ng/mL, while the LLOQ was 5.0 ng/mL.

The oral bioavailability of the NLC5 was calculated from the area under the curve (AUC) using the following equation:

$$\:\%Oral\:Bioavailability=\frac{AUC\:\left(Oral\right)\:X\:Dose\:\left(IV\right)}{AUC\:\left(IV\right)\:X\:Dose\:\left(Oral\right)}\:X\:100$$

### Stability studies

CBD NLC5 underwent a stability assessment at two storage conditions, 4 °C and 25 °C for three months. The formulations were stored in 20 mL glass scintillation vials wrapped with aluminum foil to avoid exposure to light, then they were evaluated for PS, DC, EE, and CBD release profile throughout the storage period.

### Statistical Analysis

Statistical analysis was performed using JASP software (JASP-0.18.1.0). Paired samples T-Test was utilized to compare CBD NLC5 during the stability studies in terms of DC, EE, PS, and CBD release. Statistical significance was determined by a p-value less than 0.05, signifying that if the p-value exceeds 0.05, there is no significant difference between each two time points.

## Results and discussion

### Screening and selection of lipids and surfactant

Compritol 888^®^ ATO and Dynasan^®^ 118 demonstrated a high load capacity for CBD in both lipids. Approximately 3.75 mg of CBD was melted and dissolved in each mg of solid lipid without any visible precipitation or recrystallization of CBD, even after a 24-hour cooling period.

Both sesame oil and castor oil were initially considered as liquid lipids for screening their ability to solubilize CBD based on literature references, highlighting their potential as lipid carriers for NLC development. Prior reports suggested sesame oil’s suitability for CBD lipid formulations, and its capability to improve CBD bioavailability and lymphatic transport [15, 34]. Castor oil was also considered in the lipid screening step due to its inclusion in the literature as a potential lipid carrier for NLC development [35, 36]. The results of the oil screening test showed that CBD exhibited higher solubility in sesame oil (40 mg CBD / mL) compared to castor oil (29 mg CBD / mL). Consequently, sesame oil was selected as the preferred liquid lipid carrier for developing CBD-loaded NLCs. Tween^®^ 80 is a non-ionic surfactant with an HLB value of 15 [37], and has been chosen as it was reported as an effective surfactant in the preparation of stable NLCs with high drug encapsulation capacity [38, 39], in addition to its previous incorporation in CBD formulations [40].

Considering the literature review and findings, Compritol 888^®^ ATO and Dynasan^®^ 118 were assessed for their compatibility and suitability with sesame oil, and Tween^®^ 80 in the formulation process of NLCs placebo formulations to examine their synergistic potential for advancing CBD-loaded NLCs formulations.

### Characterization of the NLCs placebo formulations

Preparing placebo formulations was a critical preliminary step to ensure the compatibility and stability of the chosen lipids and surfactants in the NLC system. This initial assessment identified the most suitable solid lipid for creating a homogeneous and stable NLC formulation, which is essential for the successful development of CBD-loaded NLCs. Placebo formulations were prepared using a ratio of 3:3:4 for solid lipid (Compritol^®^ 888 ATO or Dynasan^®^ 118) / sesame oil / Tween^®^ 80. This ratio was selected as a starting point because it has the highest amount determined for each ingredient. Throughout the preparation process of the placebo formulations, the temperature was maintained at 80 °C, while during homogenization, a temperature of 70 °C was set. The temperature reduction during the homogenization step was essential due to the expected heat generated during the homogenization process that may affect the NLCs stability.

The placebo formulations were visually inspected. During this assessment, it was noticed that the NLC prepared with Dynasan^® ^118 was separated following the homogenization step when mixed with sesame oil indicating their incompatibility. Hence, Dynasan^®^ 118 was excluded from the study. In contrast, the placebo formulation comprising Compritol 888^®^ ATO, sesame oil, and Tween^®^ 80 exhibited a uniform appearance without observation of larger particles or system separation. Additionally, evaluating the placebo demonstrated the formation of a homogeneous NLC with PS, PDI, and ZP values of 331.6 nm, 0.4, and − 30.61 mV, respectively. These findings supported the suitability of these ingredients within the selected ratios in the developing effective CBD-loaded NLCs.

### Characterization of CBD NLCs

**Fig. 1:**
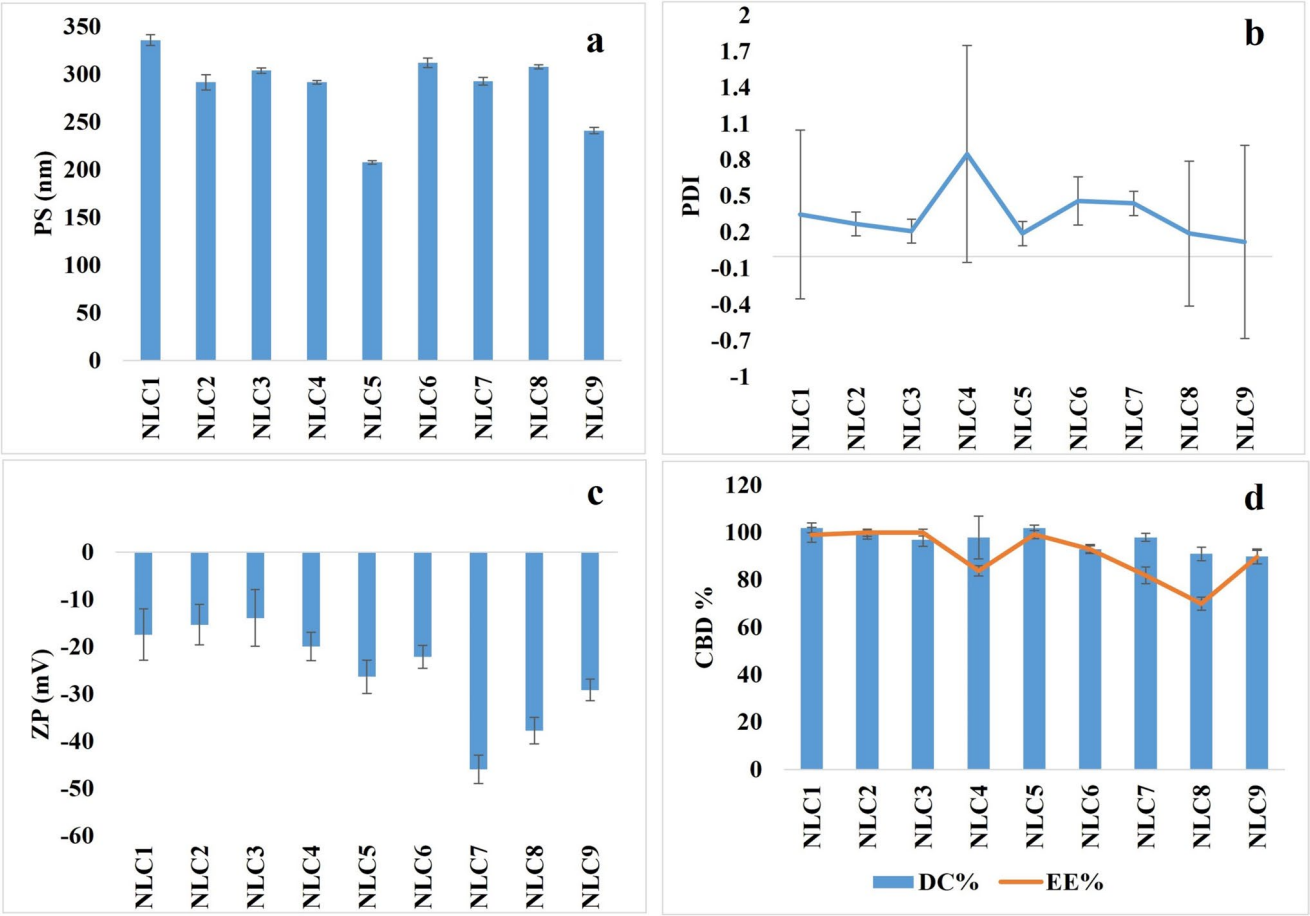
Characterization results for CBD NLCs, (**a**) Particle size (PS), (**b**) Polydispersity index (PDI), (**c**) Zetapotential (ZP), and (**d**) Drug content (DC) and entrapment efficiency (EE)

Nine CBD NLCs were prepared with different Compritol^®^ 888 ATO / sesame oil / Tween^® ^ 80 ratios and 2% CBD as shown in Table 1. Visual inspection of the prepared formulations confirmed the homogeneity of all nine CBD NLCs formulations as there were no discernible large particles adhering to the vial walls or settling at the bottom when the vial was inverted. Figure 1 presents the outcomes of the evaluation tests conducted on the CBD-loaded NLCs with the aim to identify the NLC formulation with the optimum parameters in terms of PS, PDI, ZP, and CBD EE.

The particle size of NLCs significantly influences the stability, drug release rate, and in vivo performance of the nano formulation. To achieve effective drug transport into the intestinal lymphatic system, it is favorable to maintain a small particle size [38]. Various factors, such as the type and properties of lipids and surfactants, production techniques, and processing conditions, such as temperature and time, affect the particle size of the nanoparticle formulations. Achieving a low PDI value is essential as it reflects a highly uniform and consistent size distribution which is crucial for reliable and reproducible therapeutic performance [41]. Zeta potential represents the electric charges on the particle surface, and it is a crucial parameter for predicting the physical stability of colloidal systems. Values around ± 30 mV indicate repulsive forces that contribute to the physical stability of the system [42]. The EE indicates the amount of drug encapsulated within the used lipids which is an important parameter affecting the drug release and absorption.

**Fig. 2:**
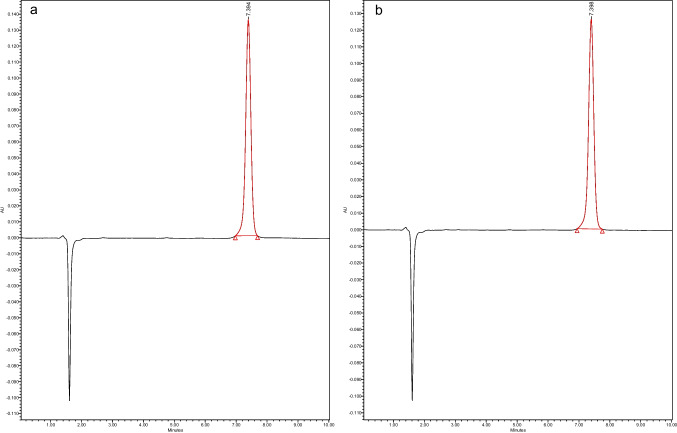
HPLC chromatogram of standard **a**) CBD at 50 µg/mL and **b**) formulation NLC5

Surfactants play an important role in stabilizing NLCs by reducing interfacial tension between lipid and aqueous phases, preventing phase separation. Thus, a higher concentration of surfactant significantly lowers the surface tension, leading to the formation of smaller-sized particles. In contrast, a lower surfactant concentration fails to sufficiently reduce the interfacial tension, resulting in the creation of larger particles [37]. Additionally, surfactants play another role in stabilizing nanoparticles by preventing them from coalescing. Thus, the surface-active properties of surfactants ensure that nanoparticles remain evenly dispersed, enhancing the overall stability and efficacy of the formulation. In this study, Tween^®^ 80 was used in the range of 2–4% w/w suggesting that increasing the concentration of the surfactant will enhance its ability to envelop the nanoparticle surfaces more effectively, thereby inhibiting their tendency to fuse or coalesce and could eventually result in improved uniformity in the PS and a decrease in aggregation [43]. The particle size of the formulations ranged between 207.7 nm and 336 nm as shown in Fig. 1a, the PDI values were between 0.12 and 0.85 (Fig. 1b), the ZP ranged from − 13.9 to −45.9 (Fig. 1c). The EE was in the range of 70–100% and DC was from 91 to 102% as shown in Fig. 1d. Figure 2 presents the HPLC chromatogram for pure CBD (50 µg/mL) and CBD in the formulation sample, NLC5.

Upon analyzing the results, it appears that the optimum surfactant concentration is below 4%. Since all the formulations with 4% w/w Tween^®^ 80 and different liquid lipid to solid lipid ratios had high particle size, 336 nm (NLC1), 291 nm (NLC2), and 304 nm (NLC3. Additionally, all the formulations with 4% surfactant had relatively less zeta potential values than formulations with 2% w/w and 3% w/w surfactant levels indicating less electrostatic repulsion between the particles. This finding supported by previous literature indicates that increasing surfactant content over a certain extent may lead to micelle aggregation, affecting system stability and reducing drug loading capacity [44].

It was also concluded that increasing the total lipid ratio in the formulation has led to the largest particle size at 4% w/w surfactant, 336 nm for NLC1, or the largest PDI at 3% w/w surfactant (0.85) for NLC4, and 2% w/w surfactant (0.44) for NLC7. This can be attributed to the increasing of the overall viscosity of the formulation, where the higher lipid contents lead to larger particle sizes and higher PDI values.

The liquid lipid-to-surfactant ratio was examined across the nine different formulations and the results indicated that a 1:1 ratio of sesame oil to Tween^®^ 80 yielded the smallest PS and PDI, particularly in NLC5 and NLC9. However, in the case of NLC9, despite achieving a small PS and low PDI, the higher proportion of solid lipid in comparison to the liquid lipid resulted in a reduction in EE (89.7 ± 5.4). The reduced EE% in NLC9 may also be attributed to the lower percentage of Tween^®^ 80, which is a recurring observation in the three formulations (NLC7, NLC8, and NLC9) that contain a lower quantity of Tween^®^ 80 (2% w/w).

Although NLC4, NLC5, and NLC6 have the same Tween^®^ 80 ratio (3% w/w), NLC5 exhibited a higher EE% which could be attributed to a higher proportion of sesame oil than Compritol^®^ 888 ATO. On the other hand, NLC4 has 1:1 ratio of sesame oil to Compritol^®^ 888 ATO and NLC6 has higher Compritol^®^ 888 than sesame oil. Previous report revealed that increasing the oil content in the NLCs formulations results in an improved drug incorporation capacity by creating more imperfections within the crystal lattice, thereby accommodating more drug loading [37]. In conclusion, it was generally observed that the formulation with optimum characteristics had higher liquid lipid to solid lipid ratio, an equivalent ratio of liquid lipid and surfactant, and a 3% of surfactant. Hence, among the various formulations, NLC5 demonstrated the lead formulation with characteristics, including the smallest particle size (207 ± 3.1 nm), a low PDI (0.199 ± 0.01), ZP of −26.3 Mv, and a notably high EE (99.23%). Hence, NLC5 was selected for further in vitro release and in vivo evaluation.

### In vitro drug release

**Fig. 3:**
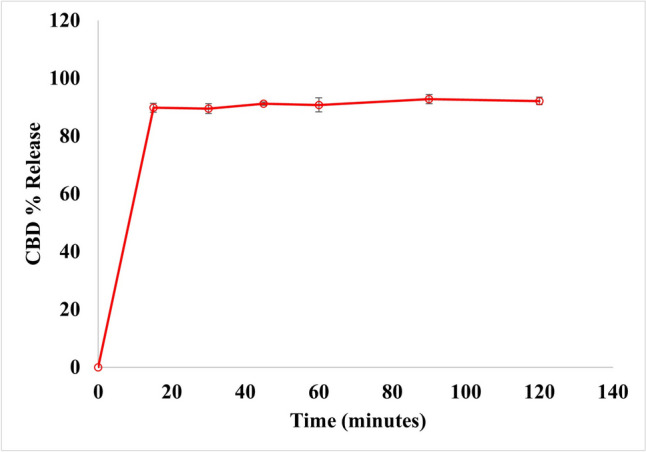
CBD release profile for NLC5 on day zero

The release studies at pH 7.4 for NLC5 in comparison with pure CBD are presented in Fig. 3. The results highlight the improvement in CBD release achieved by the developed NLC. Over 90% of CBD from the NLC5 formulation was released within 15 min, whereas the release of pure CBD was less than 1%. This finding supported the idea that NLCs formulations could generate nano-sized droplets, which create a large surface area stabilized by surfactants. This stabilization leads to faster dissolution and increased absorption, highlighting the potential of NLCs in improving the bioavailability of CBD [17].

### In vivo pharmacokinetics

To confirm the performance of NLC5 in enhancing the CBD oral bioavailability, an in vivo pharmacokinetic study was conducted in rats. The intravenous (IV) administration of CBD was used in the control group solely as a reference to calculate the oral bioavailability of CBD in the developed nanostructured lipid carriers (NLC). The IV route provides 100% bioavailability, serving as a standard to compare the systemic absorption of CBD following oral administration of the NLC formulation. The results of the analysis revealed that CBD NLC and CBD IV showed AUC of 1376.7 ng.h/mL and 1010.64 ng.h/mL respectively.

**Fig. 4:**
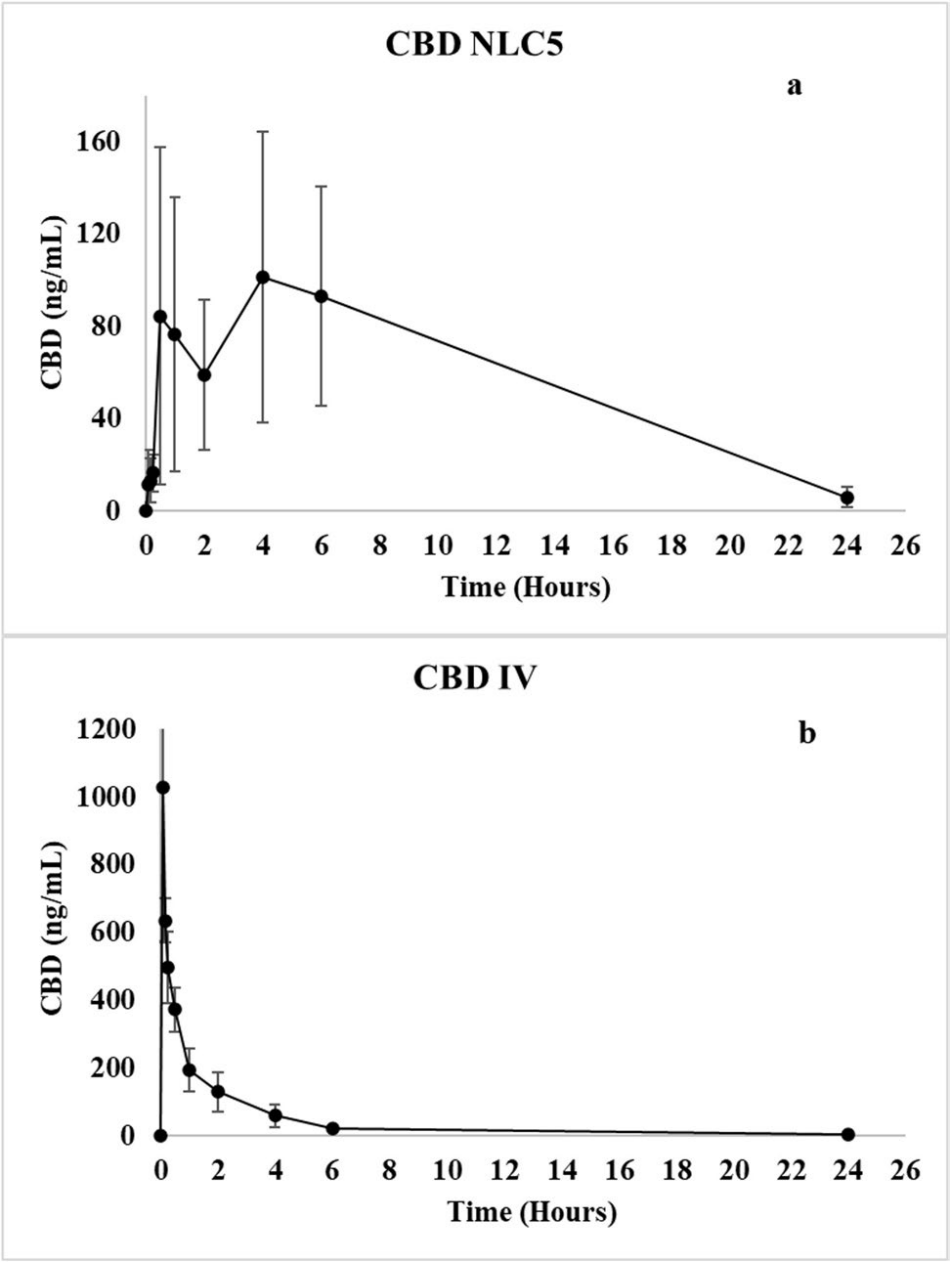
CBD plasma conc (ng/mL) after administration of (**a**) oral CBD NLC5 and (**b**) CBD IV solution in rats

Figure 4 is a graphical representation of the CBD plasma concentration-time curves following oral gavage administration of CBD NLC5 contains 5 mg CBD versus IV administration of 1 mg CBD. As shown in Fig. 4a, the formulated CBD NLCs exhibited a Cmax of 101.5 ng/mL, a Tmax, of 4 h, in contrast, the Cmax for CBD IV was 1028 ng/mL as shown in Fig. 4b.

The oral bioavailability of CBD from the NLC5 formulation was calculated to be 27% using the equation provided in the methods section which far exceeds the reported oral bioavailability of CBD, which is approximately 6% [8]. Several factors contribute to the efficacy of the NLCs in improving CBD oral bioavailability. Primarily, the increased release rate is facilitated by the small size of the lipid-based formulation, which results in a higher surface area, improved dissolution in accordance with the Noyes-Whitney equation [44]. Additionally, the change of the CBD absorption pathway from the portal vein to the lymphatic system may contribute to this observed improvement, as reported in existing literature [45]. This enhancement in bioavailability suggests that the required oral dosage of CBD could be reduced. Current reports indicate that Epidiolex doses range from 10 to 20 mg/kg administered twice daily [46]. The increased bioavailability achieved with NLC5 suggests that lower doses could achieve similar therapeutic effects, thus improving patient compliance and reducing potential side effects. These findings underscore the potential of NLC5 in optimizing CBD therapy by enhancing its bioavailability and efficacy.

### Stability studies

**Fig. 5:**
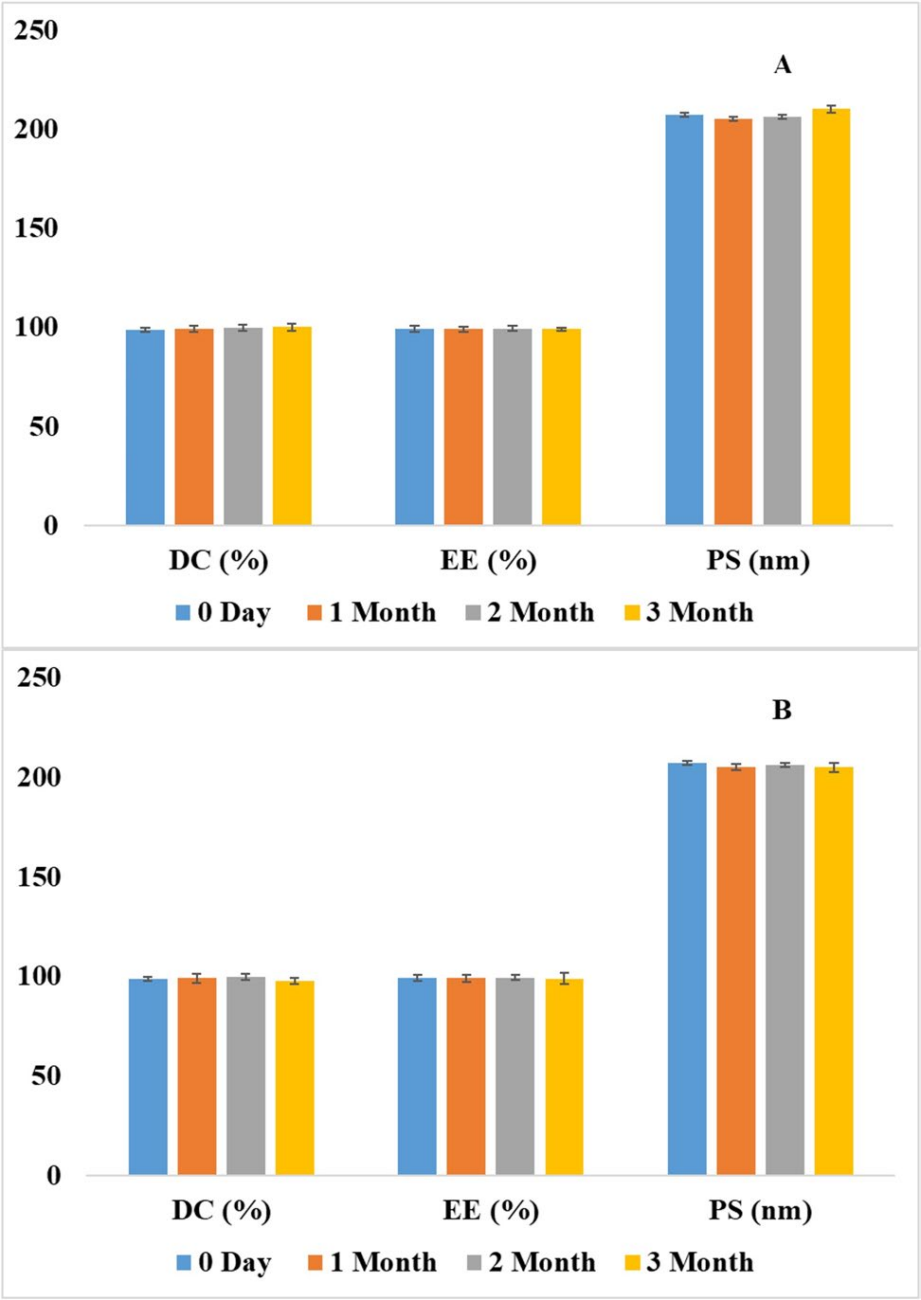
Stability results for NLC5 in terms of DC %, EE %, and PS for 3 months (0 day, 1 month, 2 month, and 3 month) (**a**) at 4 °C and (**b**) at 25 °C

**Fig. 6:**
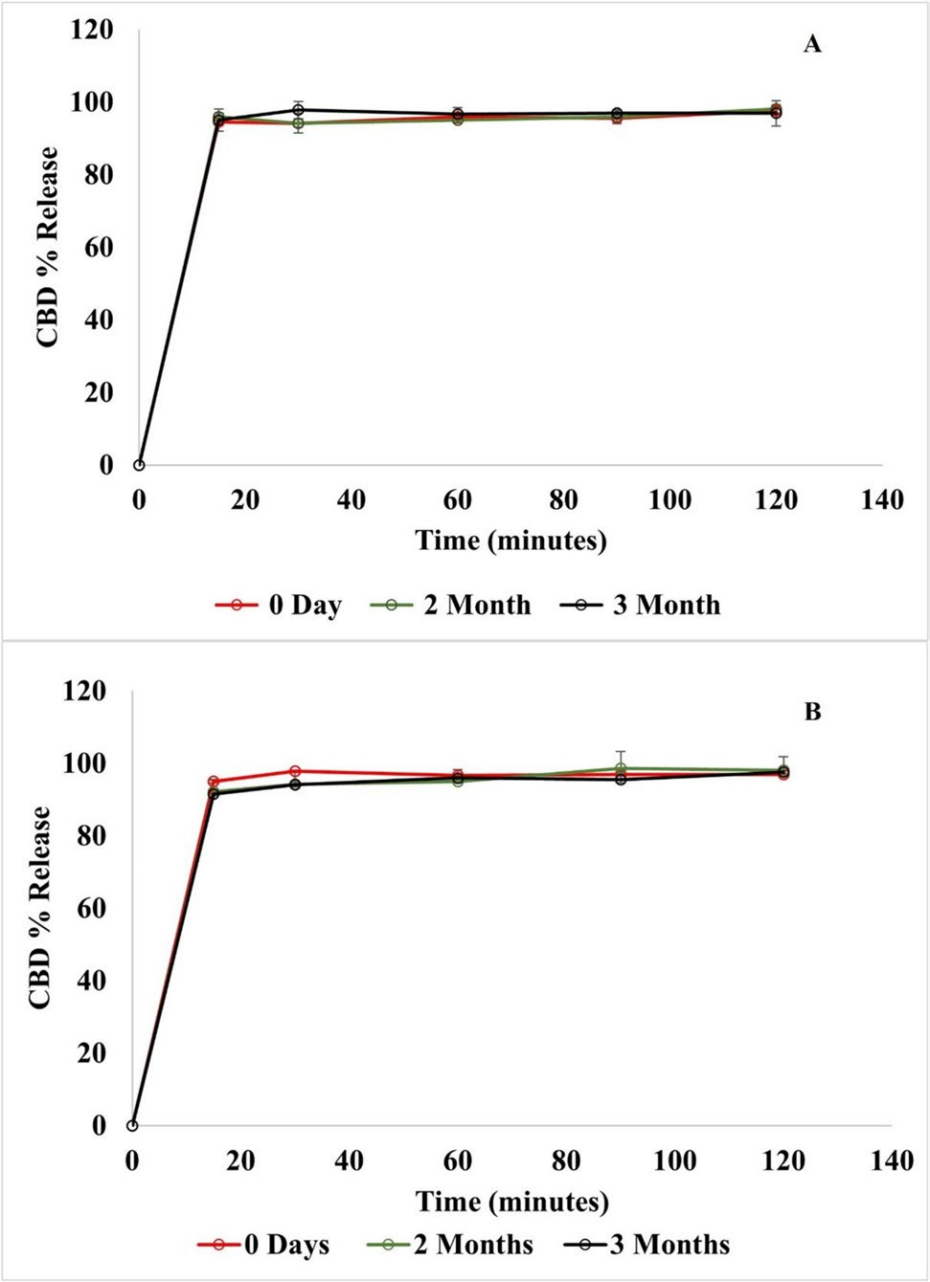
CBD release rate of CBD NLC5 at Day 0, 2 months, and 3 months) (**a**) at 4 °C and (**b**) at 25 °C

The stability assessment of the nanoparticle formulation is critical to ensuring their efficacy and quality over time. Moreover, these studies are particularly important for novel drug delivery systems like NLCs due to the potential impact of the temperature, light, and air on the stability of the nanoparticles and the encapsulated drug. The stability assessment was conducted on CBD NLC5 at two storage temperatures (4 °C and 25 °C) for a duration of three months. This study aimed to assess the stability of key parameters, including DC%, EE%, PS, and the release profile of CBD. The results indicated that CBD NLC5 maintained consistent DC%, EE%, and PS stability at both storage temperatures throughout the three months. Statistical analysis showed no significant differences (*p* > 0.05) in the different parameters between day zero result and each time point whether for the samples stored at 4 °C (Fig. 5a) or those stored at 25 °C (Fig. 5b). Additionally, the release profile of CBD from NLC5, showed no significant difference (*p* > 0.05) over the three months at both temperatures, as illustrated in Fig. 6a and b. This consistency in the release profile further confirms the stability of the NLC5 formulation under the tested conditions. In conclusion, the findings suggest that NLC5 formulation can be stored at both temperatures without compromising its efficacy.

## Conclusion

The present study successfully developed CBD NLCs to enhance the oral bioavailability of CBD using the hot homogenization method. Key formulation variables, including the selection and compatibility of solid and liquid lipids with the surfactant, were evaluated. The results indicated that sesame oil had a higher CBD solubilization capacity compared to castor oil, and Compritol^®^ 888 ATO demonstrated better compatibility with the other excipients, resulting in a uniform and homogeneous NLC formulation.

CBD NLC5 emerged as the lead formulation with smallest PS, low PDI, and high EE. In vitro release studies demonstrated an enhancement of CBD release rate. Furthermore, in vivo evaluation revealed that the oral bioavailability of CBD increased more than fourfold with the NLC5 formulation. The formulation also maintained its DC%, EE%, PS, and CBD release stability over a three-month period at both 4 °C and 25 °C storage temperatures.

These findings underscore the effectiveness of NLC formulations in elevating the oral bioavailability of CBD while ensuring stability. Additionally, this study explored an effective delivery system of CBD which could have potential clinical efficacy for the treatment of various diseases such as epilepsy, anxiety, and others.

## Data Availability

The datasets generated during and/or analysed during the current study are available from the corresponding author on reasonable request.
